# A Case Report of Recurrent Pneumothorax: A Rare Complication of Tricuspid Valve Endocarditis

**DOI:** 10.7759/cureus.46995

**Published:** 2023-10-13

**Authors:** Muhammad Bilal

**Affiliations:** 1 Internal Medicine, Merit Health Wesley, Hattiesburg, USA

**Keywords:** tricuspid valve endocarditis, bilateral chest tubes, chest tube, bilateral pneumothorax secondary to infective endocarditis, infective endocarditis

## Abstract

Intravenous drug use (IVDU) is a recognized risk factor for infective endocarditis (IE), with potential mechanisms involving direct bacterial introduction through the needle puncture. Bilateral pneumothorax, an under-reported yet significant complication of IE, was first documented in 1990. Only eleven cases of spontaneous pneumothorax (PTX) associated with septic pulmonary embolism from IE have been reported. We present a 26-year-old female with a history of IE and a prior pneumothorax. She was transferred to our facility for recurrent IE, confirmed by echocardiography and blood cultures. After an initial stable clinical course, on the fifth morning, she developed new-onset dyspnea, later diagnosed with bilateral PTX that required bilateral chest tube placement. Left-sided PTX resolved quickly, while the right-sided PTX persisted for 11 more days. Following clinical improvement, the patient was discharged on the 18th day. Promptly identifying this rare complication was crucial for the patient's survival.

## Introduction

According to a 2018 public analysis, 1.46% of the US population uses injection drugs intravenously [1]. IV drug use can have severe and wide-ranging consequences, including an increased risk of infections, such as HIV and hepatitis, as well as complications, such as skin abscesses and infective endocarditis (IE). The incidence of IE varies across the world, with reported rates ranging from 1.7 to 6.2 cases per 100,000 population per year [1]. Tricuspid valve endocarditis is commonly associated with IV drug use [2]. However, its association with bilateral pneumothorax (PTX) is uncommon and under-reported [3]. The proposed mechanism is the rupture of the subpleural emboli from the infected valve [3]. This case underscores a rare yet noteworthy complication of tricuspid valve endocarditis in a patient with an otherwise stable clinical course.

## Case presentation

We present a 26-year-old female with a history of intravenous drug use (IVDU), IE, and PTX who was transferred to Merit Health Wesley, Hattiesburg, Mississippi, for cardiology coverage due to concerns for recurrent IE. She had a prior admission with a diagnosis of IE nine months ago, which was complicated by unilateral PTX. We are unaware of the treatment she received during her previous admission. She was given intravenous ciprofloxacin, gentamicin, and vancomycin prior to arrival. On arrival, vital signs revealed a blood pressure of 125/84 mmHg, SpO2 of 97% on room air, and a respiratory rate of 22/min. Lab work done at the prior facility showed a hemoglobin level of 9.3 g/dl, a white blood cell count of 22.9 x10^9/L with 8% neutrophilic bands, a pro-calcitonin level of 2.1 Ng/mL, and a CRP level of 16.2 mg/L. Renal function tests and liver function tests were within normal limits. Table 1 mentions lab work performed at our facility.

**Table 1:** Lab values at the time of admission at our facility.

WBC	7.9
RBC	2.32
HB	6.7
HCT	20.5
MCH	29.1
MCHC	32.9
MCV	88.4
Platelet count	121
Neutrophils %	19.0 (normal reference range: 11.8-16.2)
Lymphocyte %	88.8 (normal reference range: 45.4-88.2)
Prothrombin time	18.0
INR	1.48
D-dimer	>1050 (normal reference range: 36-277)
Thromboplastin time	27.20
Sodium	137
Potassium	3.4
Blood urea nitrogen	12
Creatinine	0.88
eGfr	91
Calcium	7.5
Albumin	1.7
Alkaline phosphatase	136
ALT	49
AST	56
Total bilirubin	1.2
BNP	1200 (normal range: 0-100)
CRP (C reactive protein)	15.5
Lactic acid	1.5

**Figure 1:**
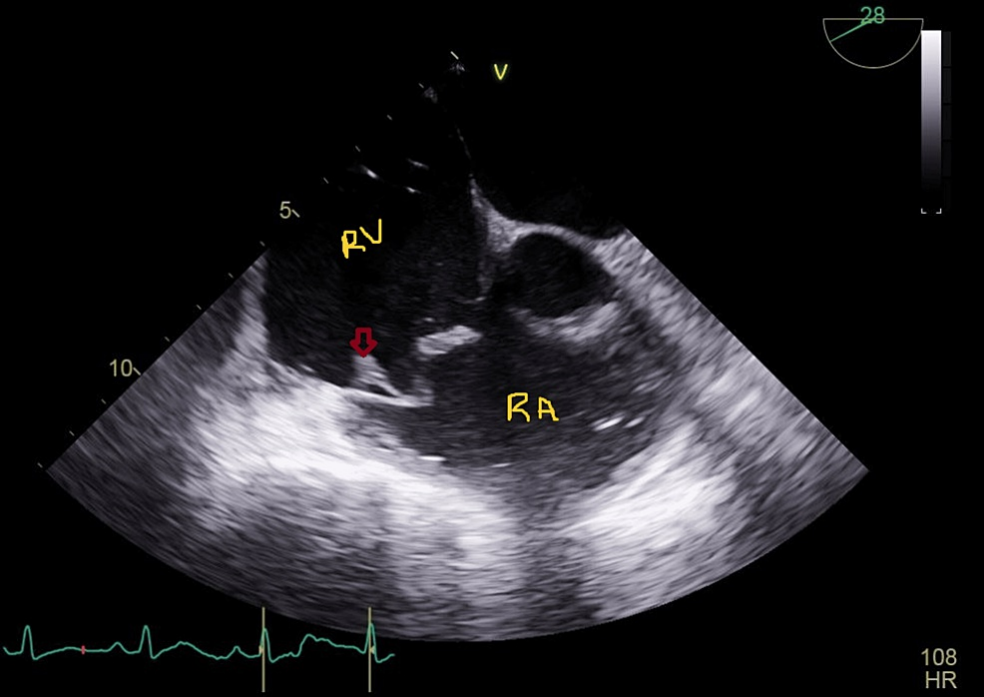
Transthoracic echocardiogram showing mobile vegetation on the tricuspid valve labeled with a red transparent arrow. The right atrium and right ventricle are labeled as RA and RV, respectively, in yellow initials. RA: Right atrium, RV: Right ventricle, HR: Heart rate

On physical examination, a 2/6 holosystolic murmur was heard over the right lower sternal border, and the patient was started on intravenous antibiotics. Blood cultures grew Staphylococcus aureus on day one, and transesophageal echocardiography revealed moderate-sized vegetations on the tricuspid valve with severe tricuspid regurgitation. We can see moderate-sized mobile vegetations on the posterior and septal leaflet of the tricuspid valve in (Figure 1).

**Figure 2:**
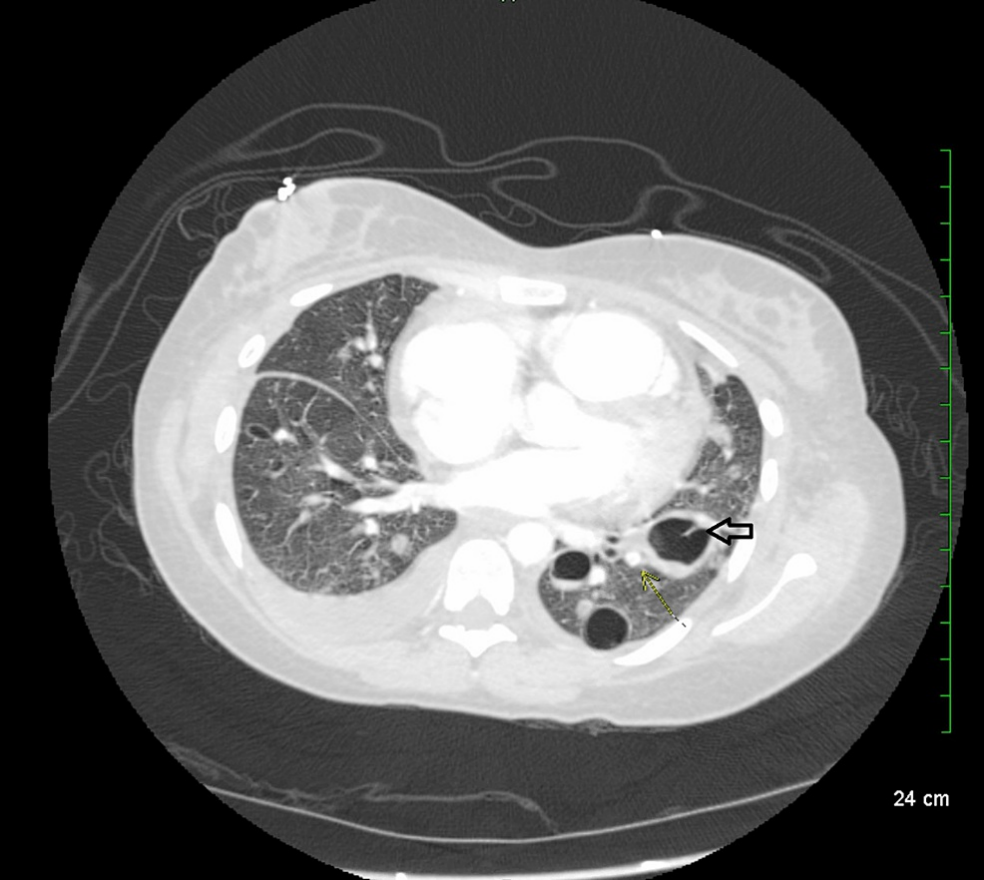
CT angiography chest on admission demonstrating multiple cavitary lesions, one of which is marked with a transparent back arrow.

We obtained a CT chest angiogram, which revealed a small, tiny non-occlusive pulmonary embolism within the posterior basal segment of the left lower lobe pulmonary artery along with multiple cavitary lesions in the left lung, as seen in Figure 2. An infectious disease specialist was consulted, and the patient's antibiotics were adjusted later as cultures speciated to methicillin-sensitive Staphylococcus aureus (MSSA), and the patient was started on intravenous oxacillin.

**Figure 3:**
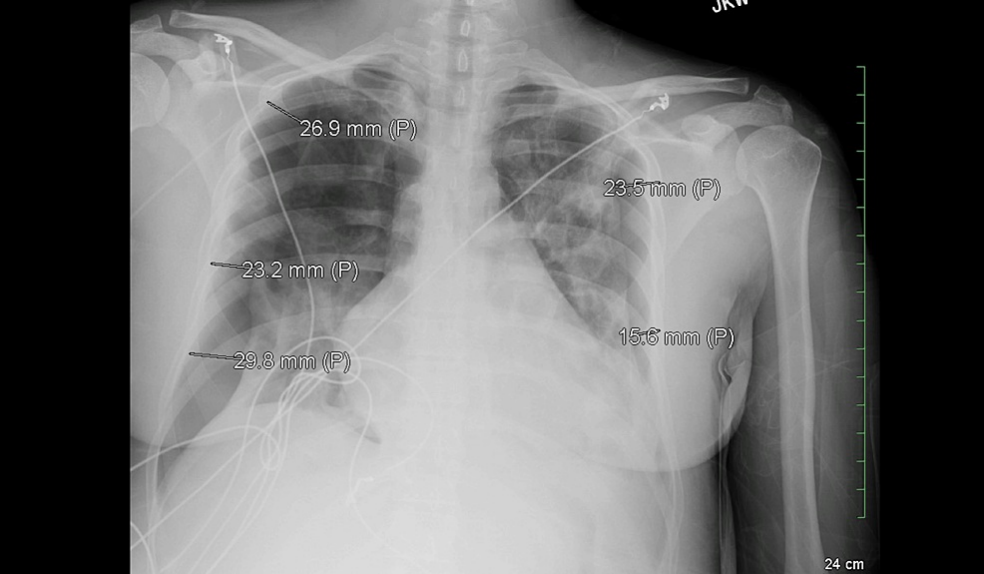
Chest X-ray showing newly developed bilateral pneumothorax. The numbered markings in the above figure show the intrapleural distance between the parietal and visceral pleura that is used by radiologists to calculate the Rhea index to classify the size of pneumothorax as small, moderate, or large [4]. This was labeled as a moderate-sized pneumothorax.

**Figure 4:**
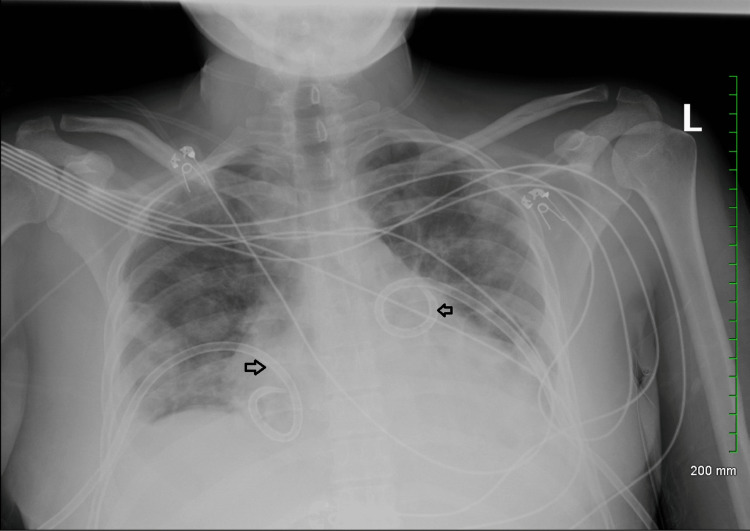
Chest X-ray showing improvement in bilateral pneumothorax after insertion of bilateral chest tubes. Bilateral chest tubes labeled with transparent arrows.

The patient developed a sudden onset of dyspnea with chills and fever on the fifth day of admission. Vitals revealed normal SpO2, and ABG revealed mild hypercapnia. She was tachycardic with a heart rate of 115, and the temperature recorded at the time was 94°F. Stat chest X-ray was obtained at the time, as seen in Figure 3, showing newly developed bilateral moderate-sized pneumothoraces, and the patient was moved to the ICU where bilateral chest tubes were placed. Repeat chest X-ray after bilateral chest tube placement is seen in Figure 4.

Left-sided PTX resolved rather swiftly, and the chest tube was removed on day three. However, right-sided PTX persisted for 11 more days. Repeat chest X-rays showed resolution of the right-sided pneumothorax, and the patient was discharged home with IV oxacillin with a peripherally inserted central catheter (PICC) on the 18th day to complete a total of six weeks of treatment from the day of the first negative blood culture. The patient was readmitted three months later for fever, chills, and concerns for suspected endocarditis; however, a repeat transesophageal echocardiogram showed no new vegetations, and small mobile vegetation was noted on the leaflet of the tricuspid valve that was significantly decreased in size as compared to prior study. Blood cultures were positive for Serratia marcescens and Enterococcus faecalis bacteremia, and the patient was discharged with a 10-day course of trimethoprim-sulfamethoxazole and amoxicillin for PICC-associated bacteremia. Of note, the patient was known to be non-compliant and had no active primary care physician or insurance at the time of admission to our facility. At discharge, multiple follow-ups were set up with a primary care physician, a cardiologist, and a cardiothoracic surgeon. However, the patient failed to comply and only followed up with cardiothoracic surgeon, and at that single appointment, the patient was recommended tricuspid valve replacement secondary to worsening right-sided heart failure. However, she failed to follow up with any appointments afterward. We are unaware if the patient had any interval interventions or imaging studies during the nine-month period between the two admissions.

## Discussion

The development of PTX is a rare complication of IE, with reported cases dating back to 1990 [5]. The proposed mechanism involves obstruction of pulmonary vessels by an embolized thrombus. Typically, this thrombus arises from an infected valve. It can be asymptomatic or lead to life-threatening dyspnea, depending on the size of vegetation and the vessels involved. These embolized thrombi can lead to the formation of cavitary lesions in pulmonary parenchyma that can later rupture and lead to the development of PTX. Common organisms include Staphylococcus aureus, coagulase-negative Staphylococcus, the streptococcal group, and the enterococcus group [6].

This case highlights the rarity of bilateral PTX in the setting of tricuspid valve endocarditis. The patient's recurrent PTX, occurring nine months apart, further distinguishes this case, as this is the only case that developed unilateral PTX with a recurrent episode of bilateral PTX nine months apart.

On review of the current literature and to our best knowledge, only 11 cases who developed PTX secondary to IE have been reported so far [3,5,7-15]. We can see in Table 1 an overview of the prior cases reported along with the number of episodes of PTX, along with oxygen supplementation status at the time of development of PTX.

**Table 2:** Case reports and studies describing pneumothorax in association with infective endocarditis.

Name of study/case report	Age/sex of the patient	Oxygen status at the time of PTX	No. of episodes of recurrent PTX	Outcome
Aguado et al. [5] 1990	19/M	Not mentioned	Nil	Discharged home
Alafify et al. [8] 2006	37/M	5L, Nasal cannula	Nil	Eloped
Yang et al. [9]2011	78/M	Not mentioned	Nil	Unknown
Sheu et al. [10] 2009	23/M	Not mentioned	Nil	Discharge home
Montano et al. [11] 2021	36/M	On ventilator	Nil	Discharge to a rehabilitation facility
Corzo et al. [12] 1992, Case 1	23/M	Not mentioned	Nil	Unknown
Corzo et al. [12] 1992, Case 2	26/M	Not mentioned	Nil	Unknown
Bhatt et al. [13] 2017	30/F	Ventilator	Two, same admission	Unknown
Dashtkoohi et al. [14] 2022	15/F	Not mentioned	Three, same admission	Discharge home
Swaminath et al. [15] 2013	25/M	Non-rebreather	Nil	Unknown
Kapoor et al. [3] 2018	33/F	Fio2 40%	Multiple, same admission	Deceased

Notably, none of the prior cases presented with recurrent pneumothoraces separated by such a lengthy interval, and, in most of the previously reported cases, patients were placed on positive pressure ventilation because of respiratory distress at presentation. However, in our patient's case, bilateral PTX developed while the patient was on room air without any supplemental oxygen.

## Conclusions

Bilateral PTX is a rare but serious complication of IE, especially in patients with a history of IVDU. Early diagnosis with urgent chest X-ray and prompt treatment with chest tube placement can avert serious sequelae and possible hemodynamic shock. Clinicians should maintain a high suspicion for PTX in stable patients with IE who develop acute onset dyspnea.
